# Income and education as predictors of return to working life among younger stroke patients

**DOI:** 10.1186/1471-2458-11-742

**Published:** 2011-09-29

**Authors:** Sven Trygged, Kozma Ahacic, Ingemar Kåreholt

**Affiliations:** 1 Department of Social Work, Stockholm University, 106 91 Stockholm, Sweden; 2 Social medicine, Department of Public Health Sciences, Karolinska Institutet, Box 170 70, 104 62 Stockholm, Sweden; 3 ARC Aging Research Centre, Karolinska Institutet and Stockholm University, 106 91 Stockholm, Sweden; 4 Department of Social Work, Stockholm University, 106 91 Stockholm, Sweden

**Keywords:** socioeconomic position, income, education, work, stroke

## Abstract

**Background:**

Socioeconomic conditions are not only related to poor health outcomes, they also contribute to the chances of recovery from stroke. This study examines whether income and education were predictors of return to work after a first stroke among persons aged 40-59.

**Methods:**

All first-stroke survivors aged 40-59 who were discharged from a hospital in 1996-2000 and who had received income from work during the year prior to the stroke were sampled from the Swedish national register of in-patient care (n = 7,081). Income and education variables were included in hazard regressions, modelling the probability of returning to work from one to four years after discharge. Adjustments for age, sex, stroke subtype, and length of in-patient care were included in the models.

**Results:**

Both higher income and higher education were associated with higher probability of returning to work. While the association between education and return to work was attenuated by income, individuals with university education were 13 percent more likely to return than those who had completed only compulsory education, and individuals in the highest income quartile were about twice as likely to return as those in the lowest. The association between socioeconomic position and return to work was similar for different stroke subtypes. Income differences between men and women also accounted for women's lower probability of returning to work.

**Conclusions:**

The study demonstrates that education and income were independent predictors of returning to work among stroke patients during the first post-stroke years. Taking the relative risk of return to work among those in the higher socioeconomic positions as the benchmark, there may be considerable room for improvement among patients in lower socioeconomic strata.

## Background

Stroke is responsible for a considerable proportion of health problems in both high and low income countries [1,2]. But while stroke is one of the leading causes of death worldwide, there has been a decline in stroke mortality in many high income countries [3]. At the same time, stroke is a leading cause of disabilities among adults, and stroke survivors often have to cope with stroke injuries that lead to both physical and cognitive impairment [4,5].

The medical and social outcomes of stroke have been examined in a number of studies. One of the most important outcomes in studies of stroke-affected persons of working age is return to work [6]. Returning to work facilitates independent living for younger stroke patients and is also likely to decrease the financial burden on society. In reviews, the proportion of post-stroke patients returning to work has varied between 11 and 85 percent [7,8]. Disability in activities of daily living (ADL) caused by stroke has been found to be the most explanatory factor for not returning to work. At the same time, studies are difficult to compare since they tend to deal with different populations, different periods, different varieties of stroke, and different definitions of work [6].

Socioeconomic position has been shown to be an important predictor of poor outcomes related to stroke. Although most studies on stroke and socioeconomic position have focused on stroke incidence and case fatality [9-13], a number of studies have examined the association between different indicators of socioeconomic position and return to work after a stroke. Studies exploring the effect of socioeconomic position based on occupation have, for example, suggested up to five times higher odds for professional managerial workers than for blue-collar workers to return to work post stroke [14,15]. In previous research, there seems to be a general understanding that socioeconomic indicators such as education, income, and social class based on occupation do play a role in returning to work [8,16-21]. However, in many of the studies the sample sizes are rather small [15,17-19] and not all studies have found significant relationships [19-23]. It also remains unclear whether different indicators of socioeconomic position predict return to work independently of each other. In one study, education was not significant when socioeconomic position based on occupation was included in the model [24]. At the same time, it has been suggested that different measures of socioeconomic position, for example education and income, should have unique explanatory power, and may be conceptualized as separate dimensions rather than being markers of a single latent variable [25]. A recent Danish study also found that both education and income were independently related to return to work after a long-term period of sick leave (all causes) [26]. Whether this also applies to return to work after stroke is unclear. Furthermore, to our knowledge no studies have examined the relationships between different socioeconomic measures, e.g. income and education, and return to work separately for different stroke subtypes.

This article aims to examine the relationship between socioeconomic position and return to working life among younger stroke patients. It analyses whether education and income have independent relations to return to work among stroke survivors, both generally and for different stroke subtypes.

## Methods

### The sample

In the study 7,081 inpatients (out of 11,864 registered) aged 40-59, who had survived a first stroke and had been discharged from a hospital in the period 1996-2000 in Sweden were analysed. These persons had not previously suffered from ischemic heart disease and had a paid work before the stroke. Linking inpatient registers to census data gave information about these individuals' level of income and their educational attainment.

### The inclusion/exclusion criteria

All major stroke subtypes were included, i.e., subarachnoid haemorrhage, cerebral infarction, intracerebral haemorrhage and 'stroke, not specified' (ICD 10 = I60, I61, I63 and I64). Due to less reliable data TIA (transient cerebral ischaemic attack, ICD 10 = G45.8 and G45.9) was excluded. To avoid possible confounding from comorbidity, people suffering from ischemic heart disease (ICD 10 = I20-I25) prior to the first stroke were excluded from the initial sampling.

To exclude student groups and young parents on leave after childbirth the lower age limit was set at age 40 (note that stroke incidence is also low before that age). The upper age limit of 59 was motivated by the fact that 65 is the official retirement age in Sweden, and the follow-up required a margin of some years before retirement.

Since we wanted to study *return *to work, only those with paid work before the stroke were included. Consequently, people who had only received some form of social security or unemployment benefit before the stroke were excluded. The inclusion criteria were based on registered income from work. The limit was set to an income from work amounting to at least € 6,600 in the year prior to the stroke. This sum represents an income comparable to approximately 25 percent of the average pay for a Swedish full-time worker in 2000. Twenty-five percent is the lowest amount of work time required to qualify for sick leave benefits in the Swedish social insurance system.

### The selection frame

**Figure 1:**
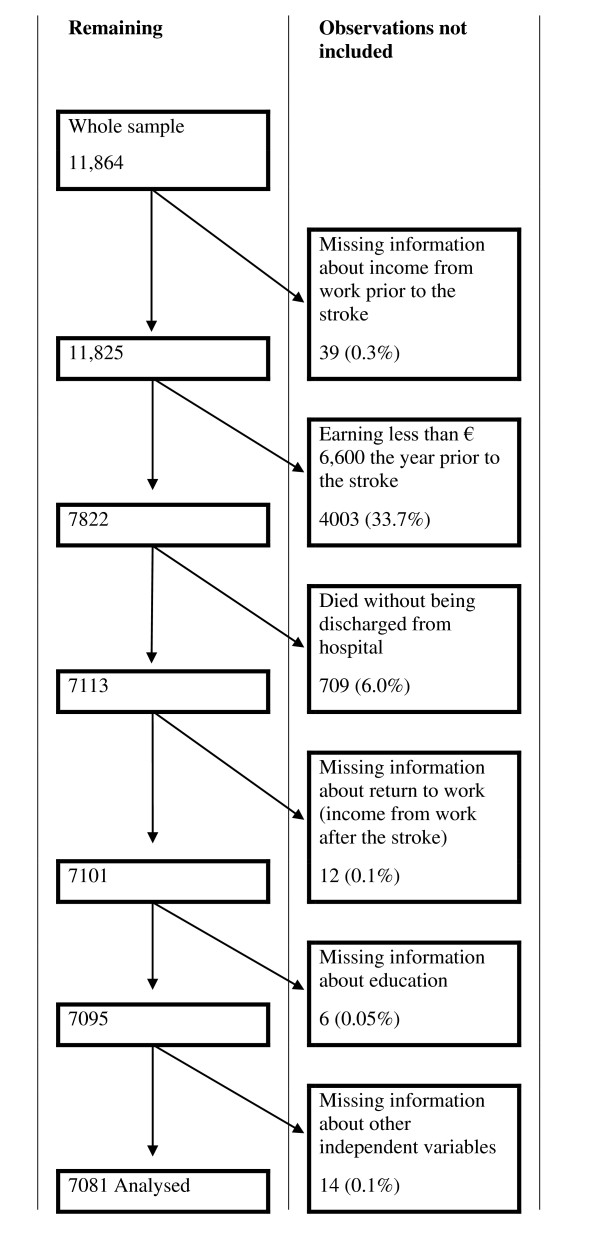
**Flow chart of how the study group was selected**.

The selected population consisted of all first time stroke patients in Sweden discharged from a hospital in the period 1996-2000 aged 40-59. The stroke patients were identified from the population register of in-patient care at the Swedish National Board of Health and Welfare and collated with a population-based data register from Statistics Sweden (SCB). The number of missing observations in the sample and the reasons to why some observations had to be disregarded is shown in the flowchart (Figure 1).

### The measures

The dependent variable, return to work, was measured in terms of patients' income from work after discharge from hospital. The lowest amount of income accepted was the same as the lowest amount accepted prior to the stroke (approximately € 6,600). Return to work implies returning to any paid job and represents a documented indicator of work capability. It was not possible to determine whether the return was to the original job or if a change of job had taken place due to the stroke

In contrast to the selection criteria for the dependent variable, the independent variable "level of income" includes all types of income - not only income from work. Level of income was divided into equally sized quartiles. Education was divided into three levels: nine-year compulsory school, upper secondary school and university studies. Since the 1950's, compulsory education in the Swedish school system has been of nine years' duration (age 7-16). Over the years, an increasing number of youths have continued their studies at upper secondary school, which means that in general, younger stroke patients have more years of education. University studies was used as the highest educational category, including those who had completed at least one semester of full-time studies; who were included since they shared more similarities with people who had longer university education in terms of return to work and mortality than those who had only completed upper secondary school.

Consequently used variables for models adjustment were age, sex, stroke subtype and length of in-patient care.

### Statistical analysis

Data were analysed with discrete time hazard regressions with time-varying baseline intensity, controlling for sex, age, age-square, days of in-patient care, days-square, and stroke subtype. The variables age and days of in-patient care were given both linear and quadratic representations to capture the curvilinear relationships.

The results are presented as relative risks (RRs). The follow-up period was four calendar years after discharge from hospital. Note that since our income data are based on calendar years, the 'first' year also includes the remaining months of the year of discharge. If stroke discharge is random over the year, this means that the average first year is 18 months (years two, three and four are each twelve months). Persons who died were assumed to die in the middle of that calendar year and be censored after half of the year. A time-varying baseline intensity was used to account for variations in return to work between the years and for the fact that the first 'year' is longer than the other years. The baseline intensity is assumed to be constant within each year [27]. The discrete time hazard regressions were performed with the STATA command STPIECE.

The results are presented in three tables. Table 1 shows descriptive statistics and Tables 2 and 3 the results from the hazard regression analyses. In Table 2 data was initially analysed with age-adjusted hazard regressions where each of the independent variables was included one by one controlling for age and age-square. Besides these analyses, three statistical models are presented. The first model included education, the second included income and the third included both education and income. In all three models, the covariables were age, age-square, sex, stroke subtype, length of in-patient care and squared length of in-patient care. Finally, in Table 3 the relative risks of returning to work are analysed separately for different stroke categories. These analyses correspond to the third model in Table 2 where education and income are entered simultaneously, controlling for age, age-square, sex, stroke type, length of in-patient care and square length of inpatient care. The p-values for the whole variables, i.e., if the variables contribute significantly to the model, were based on Wald tests.

**Table 1:** Descriptive statistics showing the proportion that returned to work after the stroke, the proportion that did not return to work, and the proportion that died.

	Returned to work		No return		P for dif return to work/no return^1^	Died without returning to work		Total
					
Independent variable	No of obs	%	No of obs	%		No of obs	%	No of obs
Sex								
Men	3241	71.3	1045	23.0	< 0.001	262	5.8	4548
Women	1626	64.2	770	30.4		137	5.5	2533
Age								
40-44	463	76.5	117	19.3	< 0.001	25	4.1	605
45-49	956	76.2	257	20.5	< 0.001	41	3.3	1254
50-54	1675	72.1	523	22.5	< 0.001	126	5.4	2324
55-59	1773	61.2	918	31.7	< 0.001	207	7.1	2898
Education								
Compulsory	1537	63.5	736	30.4	< 0.001	147	6.1	2420
Upper secondary	2060	67.4	820	26.8	0.036	178	5.8	3058
University	1270	79.2	259	16.2	< 0.001	74	4.6	1603
Income								
1^st ^quartile (lowest)	916	51.8	704	39.8	< 0.001	148	8.4	1768
2^nd ^quartile	1161	65.6	500	28.2	0.002	109	6.2	1770
3^rd ^quartile	1285	72.6	401	22.7	< 0.001	84	4.8	1770
4^th ^quartile (highest)	1505	84.9	210	11.8	< 0.001	58	3.3	1773
Days of in-patient care								
1-7	2388	80.3	465	15.6	< 0.001	129	4.0	2973
8-14	1216	73.5	355	21.5	< 0.001	83	5.0	1654
15-30	734	66.7	310	28.2	0.045	57	5.2	1101
> 30	529	39.1	685	50.6	< 0.001	139	10.3	1353
Stroke subtype								
Cerebral infarction	3293	69.3	1193	25.1	0.135	268	5.6	4754
Subarachnoid haemorrhage	776	73.8	241	22.9	0.007	34	3.2	1051
Intracerebral haemorrhage	511	57.3	305	34.2	< 0.001	76	8.5	892
Stroke, not specified (I64)	287	74.7	76	19.8	0.006	21	5.5	384
**Total**	4867	68.7	1815	25.6		399	5.6	7081

^1 ^P-values are based on χ^2^-test. Each p-value is based on the comparison between one category and all other categories combined. For instance, the p-value next to the 2^nd ^income quartile is for the comparison between the 2^nd ^quartile and quartiles 1, 3, and 4 combined.

**Table 2:** Relative risk of returning to work after stroke among all registered cases of stroke in the Swedish population between 1996 and 2000.

	Age-adjusted^a^	Model 1	Model 2	Model 3
	
Independent variable	RR^b^ (95% CI)	RR (95% CI)	RR (95% CI)	RR (95% CI)
**Education**	p **< 0.001**^c^	p **< 0.001**		p **= 0.008**
Compulsory	1 (ref)	1 (ref)		1 (ref)
Upper secondary	1.06^†^ (0.99-1.14)	**1.08** (1.01-1.15)		1.03 (0.96-1.10)
University	**1.34** (1.24-1.44)	**1.33** (1.23-1.43)		**1.13** (1.04-1.22)
**Income**	p **< 0.001**		p **< 0.001**	p **< 0.001**
1^st ^quartile (lowest)	1 (ref)		1 (ref)	1 (ref)
2^nd ^quartile	**1.39** (1.27-1.51)		**1.38** (1.27-1.67)	**1.38** (1.27-1.51)
3^rd ^quartile	**1.64** (1.51-1.79)		**1.64** (1.50-2.13)	**1.61** (1.47-1.76)
4^th ^quartile (highest)	**2.08** (1.92-2.26)		**2.02** (1.84-2.94)	**1.94** (1.77-2.12)
**Women **(ref = men, RR = 1)	p **< 0.001**	p **< 0.001**	p = 0.540	p = 0.297
Women	**0.84** (0.79-0.89)	**0.83** (0.78-0.88)	0.98 (0.92-1.05)	0.97 (0.91-1.03)
**Stroke subtype**	p **< 0.001**	p **< 0.001**	p **< 0.001**	p **< 0.001**
Cerebral infarction	1 (ref)	1 (ref)	1 (ref)	1 (ref)
Subarachnoid haemorrhage	1.03 (0.95-1.11)	**1.28** (1.18-1.39)	**1.28** (1.18-1.54)	**1.27** (1.17-1.38)
Intracerebral haemorrhage	**0.77** (0.71-0.85)	0.98 (0.89-1.08)	0.98 (0.89-1.07)	0.97 (0.88-1.07)
Stroke, not specified (I64)	1.12^†^ (0.99-1.27)	1.06 (0.94-1.20)	1.05 (0.93-1.23)	1.06 (0.94-1.19)
**Days of hospital care for stroke**^c^	p **< 0.001**	p **< 0.001**	p **< 0.001**	p **< 0.001**
Linear (per 10 days)	**0.84** (0.81-0.85)	**0.82** (0.80-0.84)	**0.82** (0.80-0.79)	**0.82** (0.80-0.85)
Quadratic/100 days	**1.004** (1.001-1.006)	**1.004** (1.002-1.006)	**1.004** (1.002-1.006)	**1.004** (1.002-1.006)

Results from discrete time hazard regressions (n = 7081).

Results from discrete time hazard regressions controlling for age and age-square. The reference category is marked "Ref". The P-value to the right of the variable name is for the whole variable, e.g. if the variable represents a significant contribution to the model. These estimations are based on Wald tests. Results in bold have p < 0.05.

Persons were excluded if they previously had had an ischemic heart disease, or TIA, or if they earned less than €6,600 in the year prior to the stroke.

^a ^In the age-adjusted models, each of the variables are presented separately, one by one, but controlling for age and age-square.

† p < 0.10.

^b ^RR = Relative Risk. A higher RR means higher probability of returning to work early. CI = Confidence Intervals.

^c ^Linear and quadratic terms are entered simultaneously. P-values indicate the combined effect of linear and quadratic representation.

**Table 3:** Relative risk of returning to work after different categories of stroke, among all registered cases of stroke in the Swedish population between 1996 and 2000.

	*Stroke subtype*		
	**Cerebral infarction**	**Subarachnoid haemorrhage**	**Intracerebral haemorrhage**
	n = 4754	n = 1051	n = 892

**Independent variable**	**RR^b ^(95% CI)**	**RR (95% CI)**	**RR (95% CI)**

**Education**	p **= **0.102	p **= **0.439	p **= **0.204
Compulsory	1 (ref)	1 (ref)	1 (ref)
Upper secondary	1.01 (0.93-1.09)	1.03 (0.86-1.22)	1.09 (0.88-1.37)
University	1.10^†^ (1.00-1.21)	1.13 (0.93-1.38)	1.25^†^ (0.97-1.60)
**Income**	p **< 0.001**	p **< 0.001**	p **< 0.001**
1^st ^quartile (lowest)	1 (ref)	1 (ref)	1 (ref)
2^nd ^quartile	**1.35** (1.21-1.50)	**1.34** (1.10-1.64)	**1.69** (1.27-2.24)
3^rd ^quartile	**1.62** (1.46-1.80)	**1.63** (1.32-2.02)	**1.72** (1.29-2.29)
4^th ^quartile (highest)	**1.94** (1.74-2.16)	**1.91** (1.53-2.39)	**2.26** (1.69-3.01)
**Women **(ref = men, RR = 1)	p = 0.746	p = 0.134	p = 0.604
Women	0.99 (0.91-1.07)	0.89 (0.76-1.04)	0.95 (0.77-1.17)
**Days of hospital care for stroke**^c^	p **< 0.001**	p **< 0.001**	p **< 0.001**
Linear (per 10 days)	**0.81** (0.78-0.84)	**0.88** (0.83-0.94)	**0.85** (0.78-0.92)
Quadratic/100 days	**1.005** (1.003-1.007)	1.000 (0.996-1.005)	1.002 (0.996-1.008)

Results from discrete time hazard regressions.

Results from discrete time hazard regressions controlling for age and age-square. The reference category is marked "Ref". The P-value to the right of the variable name is for the whole variable, e.g. if the variable represents a significant contribution to the model. These estimations are based on Wald tests. Results in bold have p < 0.05.

The same variables are included as in Model 3 in Table 2.

Persons were excluded if they had previously had an ischemic heart disease or TIA, or if they earned less than €6,600 in the year prior to the stroke. We also excluded 'stroke, not specified' (ICD 164) in Table 3 since we wanted as distinct categories as possible for the comparison.

† p < 0.10.

^b ^RR = Relative Risk. A higher RR means higher probability of returning to work early. CI = Confidence Intervals.

^c ^Linear and quadratic terms are entered simultaneously. P-values indicate the combined effect of linear and quadratic representation.

#### Procedures and ethics

In Sweden, every citizen has a personal identification number on which all registers containing personal information are based. The National Board of Health and Welfare provided information from the register of in-patient care for all persons aged 40-59 who had a first stroke in the period 1996-2000, to which Statistics Sweden then added other population data such as information on income and education. The new joint register was de-identified and the data delivered to Stockholm University. The study and the procedures were approved by the Regional Ethical Committee in Stockholm (2006/5:1).

## Results

### Descriptive statistics

A majority of 69 percent returned to work. Table 1 shows the descriptives. For example, a majority of patients were men (n = 4548, total n = 7081), and the proportion of men returning to work (71.3 percent) during the whole follow-up period of four years was higher than among women (64.2 percent). Educational and income differences are also indicated by the table. The proportion returning to work in different age groups decreases somewhat with age. Patients with a short period of in-patient care returned to work more often than those with longer periods. Cerebral infarction was the most common stroke diagnosis, but the proportion of this stroke subtype that returned to work did not differ from the average.

Patients with intracerebral haemorrhage had the longest periods of in- patient care (not shown) and returned to work less frequently (Table 1). Those with subarachnoid haemorrhage also had longer periods of in-patient care than the average (not shown) but returned to work more often (Table 1). Persons with subarachnoid haemorrhage were younger on average (51 years vs. 53 for the other stroke groups) and larger proportions were women (56 percent compared to 33 percent for other of stroke subtypes).

Average follow-up time until death, return to work or censoring was 2.4 years. As is shown in Table 1, 399 persons (5.6%) died without returning to work. Another 106 persons (1.5%) returned to work but died during the follow-up period. The average time before death in these cases was 2.5 years.

### Regression analyses

The results of the hazard regressions in Table 2 shows that people with higher education returned to work significantly more often than those with compulsory education only, and people with higher income returned to work significantly more often than people with low income. Individuals with university education were about 30 percent more likely (RR = 1.33 in Model 1) to return to work than those with compulsory education only and those in the highest income quartile about twice as likely as those in the lowest income quartile (RR = 2.02 in Model 2). When education and income were included in the same model, i.e., controlling for each other, the relative risks for education became attenuated, while the relative risks for income were about the same.

Women had a significantly lower relative risk of return to work in the age-adjusted model and in Model 1, when controlling for education, a difference which became small and insignificant when additionally controlling for income in Model 3. This suggests that men and women have about the same probability of return to work, when the gender differences in income level have been accounted for.

Results also show that, controlling for stroke subtype, there are strong associations between length of hospital care and the probability for return to work; the shorter the period of hospital care, the better the prognosis for return to work.

Those with subarachnoid haemorrhage did not differ from the other stroke subtypes (age-adjusted analyses in Table 2) with controls for age. But with additional variables included (i.e., sex, length of in-patient care, and education) those with subarachnoid haemorrhage had significantly higher probability of return to work than on average.

Table 3 shows that the association between socioeconomic position and return to work was similar for the different stroke subtypes. There were no significant differences between the different stroke subtypes (modelling not shown).

## Discussion

Our study shows that income and education predicted return to work among stroke survivors during the first post-stroke years. The results also suggest that income differences between men and women account for women's lower probability to return to work. In general, the study confirms the findings of Lindström *et al*. [28] and Howard *et al*. [14] who found significantly higher probability of return to work among persons with a higher social class. Both studies also found significant effects of education, but only in bivariate analyses. Income and education have previously been shown not only to be important factors both for identifying individuals and groups at risk of experiencing a stroke, but also for predicting different stroke outcomes; for example, if a stroke is survived, low socioeconomic position is related to higher risk of ending up in a nursing home and to a higher mortality rate [9,29,30]. To our knowledge, this is the first study that has examined return to work after stroke in a large population-based sample. Some findings from different countries and different time spans go against these findings [19-23] but it remains unclear why this is the case.

The effect of education on stroke outcome is likely to occur through pathways that involve income. Since education generally precedes income as it is completed early in life, and income is partly the result of educational achievements, education may be conceptualized as a factor underlying the later association between income and return to work. However, there must be other causes for the association between education and return to work, since both education and income, controlled for each other, were significantly associated to the probability of return to work.

Cox *et al*. [9] found that people with lower education and income tend to suffer a more severe stroke. We did find a strong association between number of days in in-patient care and return to work. While using days in in-patient care as a proxy and control for stroke severity might include confounding factors such as organisation of health care, its use can still be considered reasonable in the absence of clinical data. The analysis indicates that the relationships studied exist independently of this proxy of stroke severity.

The comparatively positive outcome for stroke survivors who had subarachnoid haemorrhage has been explored earlier and is in line with a previous finding [31]. However, there were no indications that associations between socioeconomic position and return to work differed between different stroke subtypes.

The poorer health condition of persons of lower socioeconomic status may be related to the classes' different lifestyles. For example, in one study, the socioeconomic gradient for the incidence of stroke among middle-aged persons could largely be explained by established risk factors such as smoking and alcohol consumption [12]. Moreover, in a population-based Swedish study on elderly patients with cerebral infarction [32], the socioeconomic gradient persisted when adjusted for risk factors and acute care variables. Unfortunately, since our study was based on registers there were no health behaviour data and it is not clear whether the relationships found are independent of socioeconomic differences in lifestyle.

The relationships found may be due to socioeconomic-related differences in the amount or quality of the health care received [33,34]. The findings are valid in the Swedish context, with a general health care system and far-reaching goals of health equity. Sweden is a high-income country representative of a Nordic welfare model with comparatively strong income equity [35], mandatory health care for all Swedish citizens and a rather strong emphasis on work rehabilitation. There is also a relatively low degree of inequality in regard to educational opportunity [36]. Nevertheless, even in such a context, well-educated persons (and their spouses, relatives and friends) probably have higher expectations and may be in a better position to voice their demands both for cutting-edge health care in connection with the stroke and for subsequent rehabilitation. People with higher income may also be able to pay for private care, e.g. a more elaborate rehabilitation. An OECD study including 21 countries that measured inequity in doctor utilization by income [37] found, for example, unequal physician utilization favouring better off patients in Sweden.

There are many other contextual factors to address in understanding return to work (compare, for example, Link & Phelan [38]) and there are structural differences between different sectors of the labour market. Returning to work is strongly linked to the possibility of adjustment at work, and recovery and rehabilitation measures in the work place. For example, the majority of well-educated persons have (higher paid) white-collar professions where it may be easier to find adjusted work tasks than in blue-collar professions.

In our study, men also returned to work somewhat more often and somewhat earlier than women, contrary to the findings of another recent Swedish study showing no significant sex differences in return to work [28]. Not only do men have higher incomes than women [39], labour market conditions can also be strongly contextual but work setting can also differ based on gender. For example, while there is a relatively high percentage of women in the workforce in Sweden, the country has a strongly gender divided labour market with men mostly working in the private sector and many women working in the public sector. Although our results suggest that income differences between men and women accounted for women being less likely to return to work, the reason for this may still be found in men's and women's different occupations, as well as the fact that women's jobs are generally lower paid [40].

It is also possible that well-educated and better paid people tend to find their work more stimulating, or perhaps their role in the work place is more important than for people with low pay or less education; they may be more difficult to replace and/or in a better position to voice their demands.

### Limitations

The generalisability of results to other times and countries is unclear. Since we arrived at our contribution by using population-based data, we should at least be able to give strong evidence from Sweden. Nevertheless, even for Sweden, recent policy reforms may affect the generalisability of the results over time. There has been an almost aggressive return-to-work policy in Sweden with activation measures and benefit reductions during recent years. This could possibly change the association between stroke and return to work. It is also possible that such changes do affect groups with different education and income differently.

This study is based on registers generally considered to be of high quality that provide the basic information for Swedish health care development and comparisons (see e.g. [41]). Very few cases on income and education are missing in our study. Statistics Sweden also takes active part in the methodological discussion on usage of administrative registers (see e.g [42]). Our study is also in line with the recommendations of a recent review of stroke outcomes suggesting that return-to-work studies should be based on population data and the measurement of paid work [6]. Nevertheless, there are some important limitations in our study design since we do not know the clinical condition of the stroke-affected persons. We avoided using TIA diagnosis due to less reliable data and we excluded those with prior ischemic heart disease, but there might be other co-morbidities or confounding factors due to unmeasured variables such as health behaviour and quality of care. We do not know the type of work returned to, or whether the subjects remained in the labour force after the initial phase of return. Income from work was used as a proxy for work capacity and we only studied stroke survivors who had an income from work prior to the stroke. We tried modelling other income levels (≈ €1100 and ≈ €11000). The proportion of people returning was dependent on the income level chosen, but the socioeconomic pattern remained the same (results not shown). There is also a risk of overestimating return to work in high income groups (and underestimating those in the lowest income group) because our income level was based on the average Swedish income, corresponding to 25 percent of full time work. Someone in the highest income group may work less than 25 percent, but still have an income from work equivalent to the average Swedish income.

By using population data we tried to avoid a selection bias. However, there is a potential bias from fatal events. We have no information about people who did not visit a doctor or died before reaching hospital care. The socioeconomic mortality pattern after a stroke, both before and after discharge from hospital, corresponds to the pattern of not returning to work (based on preliminary analyses from the same data, not shown). Persons with low socioeconomic position are both more likely to die and less likely to return to work. As a consequence, our results could have underestimated the association between socioeconomic position and return to work.

Since the study is based on register data, no information is available on lifestyle factors. We used all relevant covariates available. While this limitation may have resulted in omitted variable bias/unobserved heterogeneity, it was the best possible modelling given the available data.

While the highest income quartile had twice the odds of returning to work compared with the lowest quartile, those with a university education were only 13 percent more likely to return to work than those with an elementary education. This may indicate that economic resources are more decisive than educational ones (e.g. how well-informed someone is), but whether this is the case remains unclear. In the future, it would be interesting to examine more closely the determinants captured by different socioeconomic indicators.

## Conclusions

Socioeconomic position prior to a stroke predicts the chance of return to work among stroke patients during the first post-stroke years. Both education and income prior to the stroke were independent predictors of return to work. These findings underline the importance of socioeconomic position, even in a high-income country such as Sweden with relative equity in health care. The study suggests that there is a need to develop measures that facilitate return to work for persons of lower socioeconomic position, particularly those in low-income groups.

## Competing interests

The authors declare that they have no competing interests.

## Authors' contributions

ST designed the study, made the research application, acquired data and drafted the manuscript. IK has been involved in drafting the manuscript and performed the statistical analysis. KA has revised the manuscript critically for important intellectual content. All authors read and approved the final manuscript.

## Pre-publication history

The pre-publication history for this paper can be accessed here:

http://www.biomedcentral.com/1471-2458/11/742/prepub
